# Fewer Community-Acquired Colds with Daily Consumption of *Lactiplantibacillus plantarum* HEAL9 and *Lacticaseibacillus paracasei* 8700:2. A Randomized, Placebo-Controlled Clinical Trial

**DOI:** 10.1093/jn/nxaa353

**Published:** 2020-12-09

**Authors:** Irini Lazou Ahrén, Magnus Hillman, Elisabet Arvidsson Nordström, Niklas Larsson, Titti Martinsson Niskanen

**Affiliations:** Probi AB, Lund, Sweden; Department of Clinical Sciences, Lund University, Lund, Sweden; Probi AB, Lund, Sweden; Probi AB, Lund, Sweden; Probi AB, Lund, Sweden

**Keywords:** probiotic, respiratory tract infections, *Lactiplantibacillus plantarum*, *Lacticaseibacillus paracasei*, Probi Defendum^®^

## Abstract

**Background:**

Viral infections of the upper airways are the most common cause for absence from work or school, and there is evidence for probiotic efficacy in reducing the incidence and severity of these infections.

**Objectives:**

We aimed to confirm the previously reported beneficial effects of *Lactiplantibacillus plantarum* HEAL9 and *Lacticaseibacillus paracasei* 8700:2 against community-acquired common colds and identify a possible mechanism of action.

**Methods:**

In a double-blind study, healthy adults (18–70 years of age) with at least 4 colds during the last 12 months before recruitment were randomly allocated to consume either probiotics (*n* = 448; total daily dose of 10^9^ CFU with the 2 strains equally represented) or placebo (*n* = 450) once daily for 12 weeks. Recruitment took place from October to February during 2013–2016 (over 3 cold seasons). The probiotic impact on the severity of the colds (Wisconsin Upper Respiratory Symptom Survey−21) was the primary endpoint, whereas secondary endpoints included the incidence rate and duration of colds and an analysis of immune markers. Mann-Whitney U test and mixed model were used for the analysis of continuous variables and Fisher´s exact test was used for the analysis of categorical endpoints.

**Results:**

Symptom severity was not reduced after intake of the probiotic, despite the positive trend seen in the first season. However, significantly fewer colds were experienced in the probiotic group (mean of 1.24 colds) as compared to the placebo group (mean of 1.36 colds; *P* = 0.044) for subjects reporting at least 1 cold, the incidence of recurring colds was 30% lower (20.8% vs. 29.8%, respectively; *P* = 0.055), and the use of analgesics was 18% lower (26.3% vs. 32%, respectively; *P* = 0.07). After 12 weeks, the change from baseline for IFN-γ differed between the groups (mean difference of −7.01; 95% CI, −14.9 to 0.93; *P* = 0.045).

**Conclusions:**

Intake of *Lactiplantibacillus plantarum* HEAL9 and *Lacticaseibacillus paracasei* 8700:2 can be protective against multiple colds in adults prone to getting colds.

This trial was registered at clinicaltrials.gov as NCT02013934.

## Introduction

The World Health Organization defines probiotics as “live microorganisms which when administered in adequate amounts confer a health benefit on the host” (1). Their beneficial impact after oral administration begins in the gut lumen through various mechanisms, such as activation of the intestinal epithelial cells and dendritic cells or by impacting the microbial ecology and barrier function in the gut (2, 3). Probiotics are known to maintain the intestinal homeostasis and gut immunity balance and can prevent or reduce the severity of disease-associated symptoms not solely in the gut, but also at distal sites such as the airways or the brain (2, 3). Acute infectious or antibiotic-associated diarrhea, acute upper respiratory tract infections (URTIs), autoimmune diseases, stress, and bone health are only some of the areas for evaluating the benefits from probiotics (4–6). Although the levels of evidence for their benefits vary in the different health areas, probiotics are promising in preventing acute URTIs such as the common cold, especially in the more vulnerable populations of young children, athletes, and the elderly (7, 8).

On average, children have 6–8 cold episodes per year and adults have 2–4 (9). The annual cost from productivity losses, health care−related expenses, and absences from school and work due to respiratory tract infections was approximated at US$ 40 billion in the United States alone (10). Finding good preventive measures that could reduce the duration and number of common cold episodes would have an economic as well as a clinical impact (11, 12). Projections of the probiotic benefits on public health and the corresponding economic impacts have been reported for France and Canada, indicating that the usage of probiotics is promising in reducing the socioeconomic burden of acute URTIs (11, 12).

In general, probiotic efficacy is characterized by strain specificity, and contradictory results may be seen on the immunomodulatory capacity of different bacteria within a species (13, 14). Furthermore, characteristics of the studied populations, such as age or medical history, the length of the intervention, or the product formulation may also add to the factors that can affect the efficacy of the probiotic. *Lactiplantibacillus plantarum* HEAL9 and *Lacticaseibacillus paracasei* 8700:2 have been used in several studies of immune effects, either as monostrains or in combination and in blends with other bacteria (15–17). In a placebo-controlled intervention study, *L. paracasei* 8700:2 significantly induced the phagocytic capacity of white blood cells in healthy adults and increased the number of natural killer (NK) T lymphocytes within 2 weeks of intake (17). This was interpreted as a priming of the immune system that could be beneficial, for example in the prevention of viral infections. In the studies by Berggren et al. (18) and Busch et al. (19), the combination of *L. plantarum* HEAL9 and *L. paracasei* 8700:2 was found to reduce the incidence, severity, and duration of common cold episodes. The same combination of strains was also shown to be of benefit against self-reported cold infections in a population of Swedish children aged 1–6 years who were attending day care (20).

The present study was undertaken to confirm the previously reported results on the efficacy of *L. plantarum* HEAL9 and *L. paracasei* 8700:2 against the common cold in healthy adults. The current study design included a more controlled evaluation of the incidence and severity of the cold episodes compared to the previously conducted studies with the same combination of bacteria. The start of a cold had to be confirmed by a physician, and the validated Wisconsin Upper Respiratory Symptom Survey−21 (WURSS-21) and Jackson scale were used for the daily assessments of the duration and severity of cold symptoms (21, 22).

## Methods

### Design of the study

The study was randomized, double-blind, and placebo-controlled, with the objective of evaluating the benefit of *Lactiplantibacillus plantarum* HEAL9 (DSM 15312) combined with *Lacticaseibacillus paracasei* 8700:2 (DSM 13434; Probi Defendum^®^, supplied by Probi) in subjects with increased susceptibility to the common cold. It was a multicenter trial conducted at 14 sites in Germany (ClinicalTrials.gov ID: NCT02013934), and ethical approval was received by the corresponding local Ethics Committees in Berlin, Frankfurt am Main, Münster, Hannover, and Dresden. All subjects provided signed informed consent before randomization into 1 of the 2 study groups. The recruitment was initiated in October 2013 and was completed in February 2016 during the 3 common-cold seasons of 2013–2014, 2014–2015, and 2015–2016. In each season, study participants were recruited from October until February, and each participant was included in the study only during 1 season. This clinical study was performed in compliance with the Declaration of Helsinki, as well as the ICH-GCP guidelines and European Union recommendations (CPMP/ICH/135/95).

### Study participants

Healthy men and women (18–70 years of age) with susceptibility to the common cold (a minimum of 4 colds in the last 12 months) were eligible for inclusion in the study provided they agreed to refrain from major changes in their diet and physical activity and committed not to use any products that might influence the study outcome (pharmaceuticals or botanicals). Intake of “rescue medication” was allowed and was restricted to paracetamol (maximum 2 g/day), decongestant nose drops or nasal spray (isotonic sea water), or antibiotics (if deemed necessary by the health-care provider). The exclusion criteria at randomization included acute or chronic disease in the airways or gut, a history of nasal reconstructive surgery, the presence of nasal ulcers/nasal polyps or other conditions that could cause nasal obstruction, congenital or acquired immunodeficiency disease, Bechterew's disease, a body temperature above 37.5°C, suspected swine flu or influenza, vaccination with an adjuvanted vaccine within 3 months or a nonadjuvanted vaccine within 6 weeks prior to the study start, serious organ or systemic diseases, sleep disorder, psychiatric disorders, known sensitivity to the ingredients of the investigational product, any allergic reaction or regular intake of products that might influence the study outcome (e.g., immune suppressants/immune stimulants, including paramedication, such as Echinacea, analgesics/antirheumatics, antiphlogistics, antitussives/expectorants, influenza remedies, mouth or throat therapeutics, decongestants, antibiotics, antihistaminergic drugs, probiotics) within the last 4 weeks prior to the study start, habitual usage of nasal drops/spray, pregnancy or nursing, alcohol or drug abuse, simultaneous participation in another clinical trial, or participation in a clinical trial within the last 30 days. Usage of other products with functional food or dietary supplements containing live bacteria cultures was not allowed.

### Study procedures

The study was advertised in the local communities and all study visits took place at the clinics of the recruiting practitioners. The eligibility of the study participants was confirmed at a screening visit (Visit 1) after a physical examination and documentation of the medical history and concurrent medication. The screening visit also included a routine laboratory analysis of venous blood for clinical chemistry (creatinine, aspartate transaminase, alanine transaminase, gamma-glutamyl transpeptidase, alkaline phosphatase) and hematology (hemoglobin, hematocrit, erythrocytes, thrombocytes, and leukocytes), as well as an analysis of urine samples by dipstick (glucose, protein). A new visit (Visit 2) was scheduled 1–5 days later for the randomization of the eligible study participants into 1 of the 2 study groups. Each subject was given a study diary to be filled in daily during the intervention period. For each day in which the subjects answered “yes” to the question “do you think you have a cold?” or “do you think you are coming down with a cold?,” they also had to fill in 2 additional questionnaires: the Jackson scale (22) and the WURSS-21 (21). Visit 3 was scheduled approximately 14 days after Visit 2 and Visit 4 was at the end of the 12-week intervention period. Additional visits were scheduled upon the occurrence of cold episodes (episode visits) and included a physical examination for the confirmation of the colds and the collection of nasal swabs for viral analysis. Any adverse event (AE), intake of any concomitant medication, or a change in the concurrent medication reported at Visit 1 was registered at each study visit. A telephone contact halfway through the intervention period was also included in the study design to remind subjects about the daily intake of the study product and the filling in of the study diary. Furthermore, additional blood sample analyses were done for a subset of approximately 90 participants in the first common cold season (2013–2014). Blood samples were taken at Visits 2 (baseline) and 3 (14 days after start of intervention) for the flow cytometry analysis of NK cells (CD3-CD16+, CD56+), invariant NK T-cells (iNKT) (CD3+Va24:Ja18+), T helper lymphocytes (CD3+CD4+), cytotoxic T cells (CD3+CD8+), and B cells (CD3-CD20+). CD4+ and CD8+ cells were also analyzed for the activation markers HLA-DR, CD25, CD45R0, CD45RA, and Foxp3 (only for the CD4+ cells). In addition, from the same subset of the study population, blood samples were collected at Visits 2, 3, and 4 for storage of serum and plasma aliquots at −80°C. The U-PLEX Human Assays platform by Meso Scale Discovery (MSD^®^) was used for the analysis of IFN-γ, TNF-α, IP-10, TRAIL, IFN-α2a, IL-2Rα, and fractalkine in serum. The analysis was done according to the instructions by the manufacturer.

### Definition of a common cold episode

The start of a common cold episode was defined by the following 3 criteria, which had to be met by the subjects at least 2 days in a row: *1*) answering "yes" to either "do you think you have a cold?" or "do you think you are coming down with a cold?"; *2*) reporting at least 1 of the 4 cold symptoms: nasal discharge (runny nose), nasal obstruction (plugged or congested nose), sneezing, or sore (scratchy) throat; and *3*) scoring at least 2 points on the Jackson scale (22).

The Jackson score was calculated by adding up the following 8 symptom scores: sore throat, blocked nose, runny nose, cough, and sneezing (local symptoms), as well as headache, muscle ache, and chilliness (systemic symptoms). Symptoms were assessed on a 4-point scale: 0 = none (symptom not present in previous 24 h), 1 = mild (present, but not disturbing or irritating), 2 = moderate (symptoms sometimes disturbing/irritating), and 3 = severe (symptoms disturbing/irritating most of the time).

In addition, a cold episode had to be confirmed by the investigator during the episode visit. The end of an episode was defined as the last day with symptoms that was followed by 2 symptom-free days. Further, any viral infection with positive results for the presence of influenza virus was not considered in the analyses of the common cold episodes.

### Study product

The active study product consisted of Probi Defendum^®^, which is a combination of the 2 probiotic bacteria *Lactiplantibacillus* (previously named *Lactobacillus) plantarum* HEAL9 (DSM 15312) and *Lacticaseibacillus* (previously named *Lactobacillus*) *paracasei* 8700:2 (DSM 13434). Each bacterial strain was equally represented in the total dose of 1×10^9^ CFU/day. The placebo was of identical appearance, taste, and texture as the active product, excluding the bacteria. The study product was supplied in sachets containing a powder with freeze-dried bacteria and maltodextrin as filler. The powder was to be dissolved in 100 ml of water or another cold drink and consumed once daily for 12 weeks, preferably at breakfast. The study participants were randomly allocated to receive the probiotic product or placebo based on a randomization list with blocks of 4 and the ratio of 1:1 of the active probiotic product to placebo (i.e., they were given the next available randomization number from the list). The randomization list was generated by an independent statistician using the randomization scheme BiAS V 9.2 (2009) with no stratification by site. Sealed envelopes were prepared for the allocation concealment and were safely stored by the investigators throughout the study. The labelling of the study product and the preparation of the sealed code envelopes were done by personnel not otherwise involved in any study-related activities. Both study participants and investigators were blinded to the identity of the study product.

### Outcomes

The primary objective of the study was to show the benefit from using *L. plantarum* HEAL9 and *L. paracasei* 8700:2, as compared to placebo, on the severity of cold episodes experienced during the intervention period of 12 weeks, as measured by WURSS-21. The secondary endpoints included the evaluation of the impact of the probiotic in comparison to the placebo on the incidence, frequency, and duration of the episodes; the incidence/frequency of recurrent episodes; the usage of concomitant/rescue medication; and the safety/tolerance of the product. The assessment of the secondary endpoints was based on the information provided by the participants and the investigators in the study-related documents (diary, Jackson scale, WURSS-21, and case report forms).

### Sample size

The number of cold episodes was used as the statistical unit for the estimation of sample size. Using previously reported data by Busch et al. (19) from a study that was also conducted in Germany, the sample size estimate was based on an expected difference of 40 severity score points and an overall standard deviation of 100 points (Cohen's effect size, 0.4). Based on the expected difference in severity score between the groups, 106 cold episodes per group were required to comply with the nonparametric testing of a group difference at a 4.75% level of significance (2-sided) and a power of 80%. Assuming that at least 50% of the subjects would have at least 1 cold episode and would have 1.4 episodes on average, and taking into account that the common cold episodes were expected to occur in an unbalanced manner (30% more cold episodes in the placebo group compared to the probiotic group), it was concluded that 312 subjects were expected to report 216 cold episodes in total. At the end of the cold season in 2013–2014, 249 subjects had been randomized into the study and reported 157 valid cold episodes. A predefined interim analysis of the primary endpoint and the evaluation of the results by an independent data monitoring committee (IDMC) resulted in the extension of the recruitment period. The sample size was reestimated and the requirement for a total of at least 453 valid cold episodes resulted in the continued randomization of participants until February 2016. The interim analysis was conducted by a statistician not involved in the final analysis of the data.

### Statistical analysis

The statistical analysis was performed using the SAS software package, version 9.4. The nonparametric Mann-Whitney U test was applied for the analysis of continuous variables, whereas Fisher's exact test was used for the categorical endpoints. For the analysis of the primary endpoint, the AUC over the daily WURSS-21 scores (as documented in the subject diary) for all days of a cold episode was calculated by the trapezoidal approximation. In cases of days with missing WURSS-21 summary scores in between 2 consecutive days with nonmissing WURSS-21 summary scores, missing value imputation using linear interpolation was performed. For missing WURSS-21 summary scores at the start day(s) or the stop day(s), each missing WURSS-21 item was replaced by the median item score of the first or last day where the WURSS-21 score could be calculated. The predefined main analysis set in the study was the intent-to-treat (ITT) population that had reported valid cold episodes. The term “cold episode” will hereafter refer to “valid” cold episodes reported in the study. For the exploratory analysis of the primary endpoint, using the individual as the statistical unit, mean daily WURSS-21 summary scores were calculated without using the AUC. The ITT population was used for this exploratory analysis and for the analysis of concomitant medication use and adverse events (i.e., all participants and not only those with valid colds). An ANOVA was applied for the sensitivity analysis evaluating the interaction effect of the fixed effect variables “study group” and “season.” Cohen's d effect size was calculated using an online calculator (https://campbellcollaboration.org/research-resources/effect-size-calculator.html). A mixed model was used for the analysis of serum markers measured with the U-PLEX platform and Wilcoxon signed-rank test was applied for the analysis of the changes from baseline within each group. For the analysis of the primary endpoint, a *P* value <0.0475 was considered significant (alpha adjustment was used to account for multiple testing at the interim and the final analysis), whereas a *P* value <0.05 was considered significant for all other analyses. Data are shown as means ± SDs if not otherwise indicated.

## Results

**FIGURE 1:**
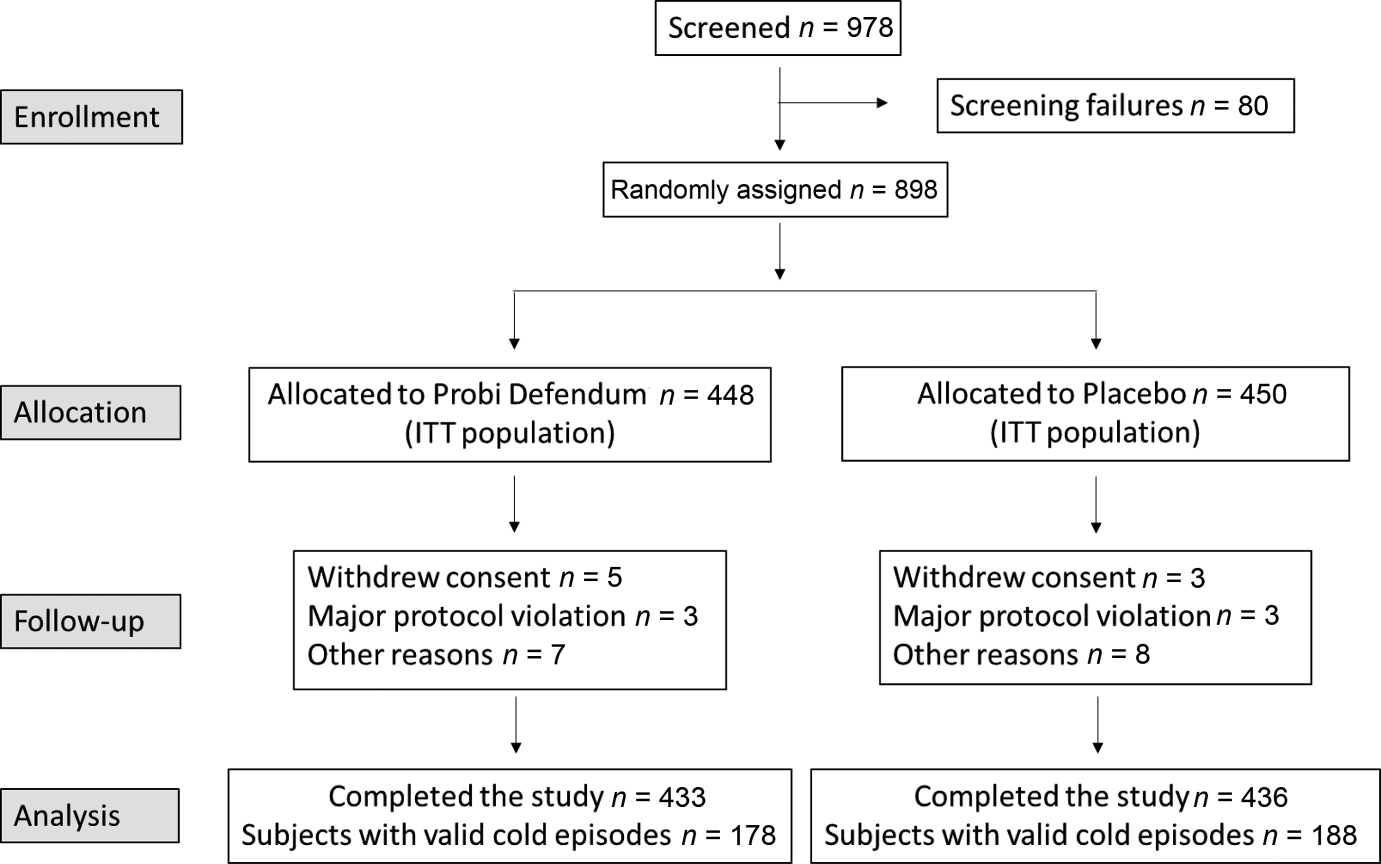
Participant flowchart. Abbreviation: ITT, intent to treat.

A total of 898 subjects out of the 978 screened were randomized into the study (**Figure 1**) to consume either the probiotic product (*n* = 448) or the placebo (*n* = 450). There were 249 subjects randomized in the first winter season (2013–2014) and 649 more subjects randomized in the following 2 winter seasons after the interim analysis and reestimation of sample size. The drop-out rates in the probiotic group (15/448; 3.3%) and the placebo group (14/450; 3.1%) were comparable. There were no differences in age, gender, or weight of the subjects between the 2 study groups, as presented in **Table 1**, nor were there differences in medical history or usage of concurrent medications prior to the study start. Most of the study subjects were in the age range of 20–54 years old (79.2% in the probiotic group and 77.3% in the placebo group). There was good compliance for intake of the study product, with a mean of 99.1% in the probiotic group and 98.9% in the placebo group. None of the safety laboratory parameters assessed at screening had values of clinical significance, and the results were similar in the study groups.

**TABLE 1:** Baseline characteristics of the 898 study participants enrolled in this randomized controlled trial

	Probiotic, *n* = 448	Placebo, *n* = 450
Age, y	40.6 ± 13.5	40.8 ± 14
Gender		
Females (%)	282 (62.9)	294 (65.3)
Males (%)	166 (37.1)	156 (34.7)
Weight, kg	74.3 ± 17	73.7 ± 16.1
BMI	25.0 ± 4.9	25.0 ± 4.9

Values are mean ± SD or frequency (%).

### Severity of cold episodes

The severity of the reported cold episodes, based on data from the WURSS-21 questionnaires, did not differ between the groups. The mean severity score, measured as AUC_WURSS-21_ for the cold episode days, was 321 ± 283 AUC units based on 221 episodes reported in the probiotic group and 301 ± 285 AUC units based on 255 episodes reported in the placebo group (*P* = 0.69). A predefined interim analysis with the data from the first cold season (2013–2014) showed a reduced severity score of the reported colds and a trend for benefit from using the probiotic product (244 ± 212 AUC units) compared to using the placebo (324 ± 339 AUC units; *P* = 0.10). The results from this analysis were evaluated by an IDMC that recommended the continued recruitment of participants until the number of cold episodes aimed for had been obtained. The results from the post−interim analysis phase (i.e., cold seasons 2014–2015 and 2015–2016) did not differ between the probiotic group (351 ± 301 AUC units) and the placebo group (288 ± 248 AUC units; *P* = 0.98).

**FIGURE 2:**
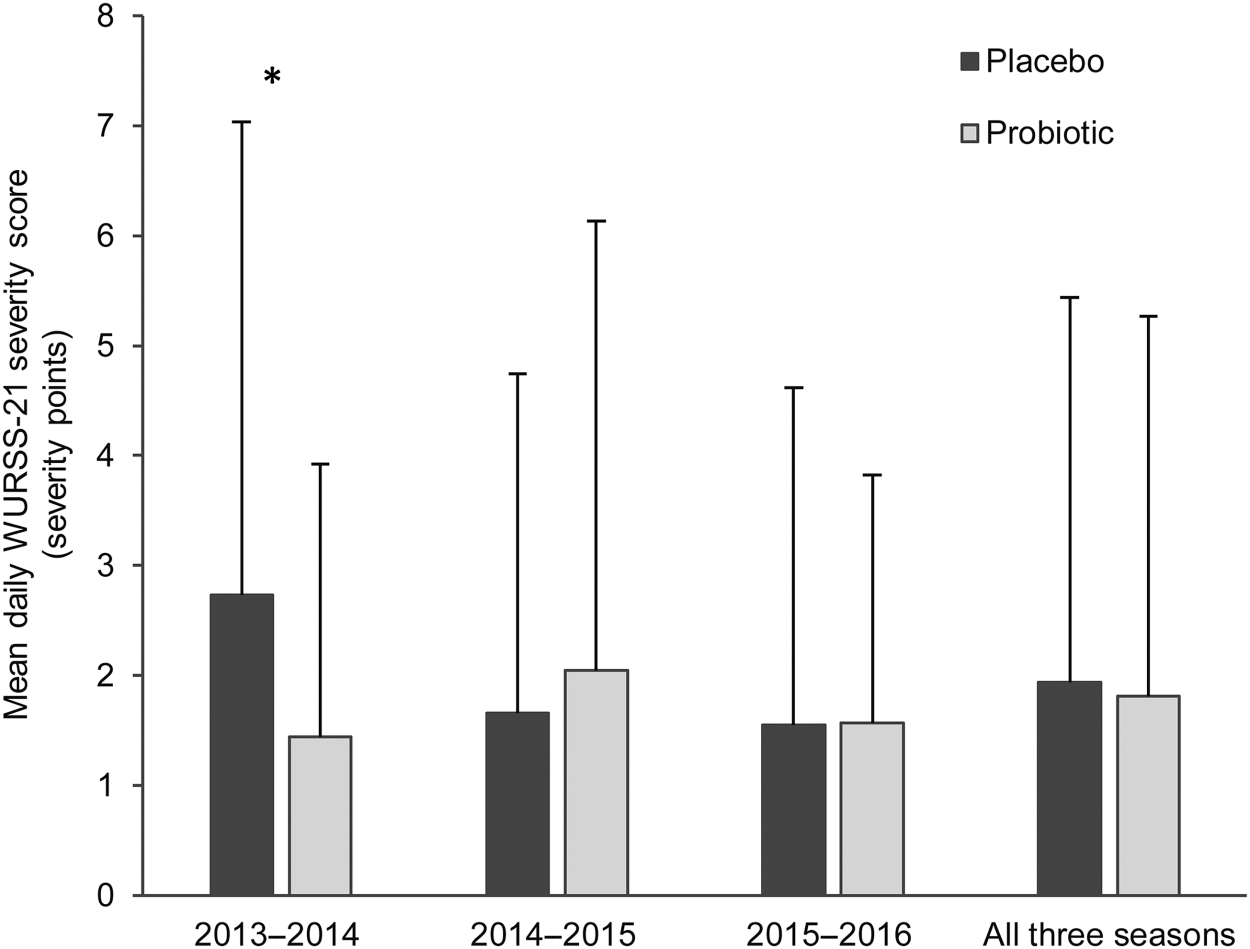
Mean daily WURSS-21 symptom score in the analysis, including all subjects randomized in the study and in each of the 3 seasons separately. Subjects without valid colds contribute with a severity score equal to “0.” Data represent means ± SDs. A Mann-Whitney U test was performed to detect differences between the 2 groups. Probiotic group: 2013–2014, *n* = 123; 2014–2015, *n* = 255; and 2015–2016, *n* = 70. Placebo group: 2013–2014, *n* = 126; 2014–2015, *n* = 254; and 2015–2016, *n* = 70. *P < 0.05. Abbreviation: WURSS-21, Wisconsin Upper Respiratory Symptom Survey−21.

An exploratory analysis of symptom severity was conducted using the individuals as the statistical unit. Subjects not reporting any valid common colds were included in the analysis and contributed with a daily severity score of 0 points. As presented in **Figure 2**, there was a significant difference in mean daily WURSS-21 score in the probiotic group (1.44 ± 2.48) compared to the placebo group (2.73 ± 4.31; *P* = 0.019) during the first cold season but not in the other 2 seasons. The mean daily WURSS-21 score did not differ between the probiotic group (1.81 ± 3.41) and placebo group (1.94 ± 3.50; *P* = 0.48) when analyzed for all 3 seasons together. The difference between the seasons with regards to the severity score based on WURSS-21 was also confirmed by an analysis of the interaction effect between “study group” and “season” using ANOVA (P = 0.007; data not shown).

The results obtained with WURSS-21 were further in line with the mean daily severity score reported based on the Jackson scale, with a significant difference between the probiotic group (0.275 ± 0.450) and the placebo group (0.482 ± 0.679; *P*= 0.014) in the first cold season. When analyzed in all 3 seasons, the severity score from the Jackson scale did not differ in the probiotic group (0.313 ± 0.545) and the placebo group (0.346 ± 0.581; *P* = 0.47).

Furthermore, since a difference was identified between the probiotic product and the placebo with regards to the severity score in cold season 2013–2014, a separate analysis of the cold-specific items in WURSS-21 was also conducted. These items described the nasal, pharyngeal, and bronchial symptoms, which were less severe in the probiotic group compared to the placebo group (*P* = 0.028) in the cold season of 2013–2014 (**Table 2**).

**TABLE 2:** Mean daily Wisconsin Upper Respiratory Symptom Survey−21 symptom severity score for subjects participating in the study during the season 2013–2014

Symptom group	Probiotic, *n* = 123	Placebo, *n* = 126	*P*
Sum of WURSS-21 score	1.44 ± 2.48	2.73 ± 4.31	0.019
Sum of cold symptoms	0.589 ± 0.972	0.994 ± 1.497	0.028
Nasal	0.283 ± 0.461	0.490 ± 0.715	0.024
Pharyngeal	0.220 ± 0.402	0.368 ± 0.580	0.045
Bronchial	0.101 ± 0.180	0.177 ± 0.328	0.10

Values are means ± SDs. All subjects in the ITT population during season 2013–2014 were included in the analysis. Subjects without valid cold episodes (validity of the colds is defined in Methods) contributed with a symptom severity score equal to “0.” A Mann-Whitney U test was used for the comparison of the 2 study groups. Abbreviations: ITT, intent to treat; WURSS-21, Wisconsin Upper Respiratory Symptom Survey−21.

### Number of cold episodes

Valid cold episodes occurred in 178/448 (39.7%) subjects in the probiotic group with 221 episodes in total and in 188/449 (41.9%) subjects in the placebo group with 255 episodes in total ( *P* = 0.54). The number of valid cold episodes per subject ranged between 1 and 4, with a mean number of 1.24 ± 0.51 episodes in the probiotic group and 1.36 ± 0.60 episodes in the placebo group (*P* = 0.044) in those who had at least 1 cold. This significant difference between the 2 groups was in line with a lower incidence of recurring cold episodes in the probiotic group, with 37/178 (20.8%) of the subjects reporting more than 1 episode compared to 56/188 (29.8%) subjects in the placebo group (*P* = 0.055). A significantly lower incidence of colds was identified in the probiotic group compared to the placebo group during the first cold season, with 50/123 (40.7%) and 67/125 (53.6%) participants, respectively, reporting at least 1 cold (*P* = 0.043). In the following 2 seasons (2014–2015 and 2015–2016), there was no difference between the groups, with 128/325 (39.4%) participants in the probiotic group and 121/324 (37.3%) in the placebo group reporting at least 1 cold.

### Duration of cold episodes

The duration of cold episodes ranged between 2 and 37 days, with a mean duration of 8.0 ± 3.9 days (*n* = 221 episodes) in the probiotic group and 8.0 ± 4.2 days (*n* = 255 episodes) in the placebo group (*P* = 0.51). The results were similar in the first winter season, with a mean duration of 7.4 ± 3.4 (*n* = 62 episodes) days in the probiotic group and 8.2 ± 4.9 (*n* = 95 episodes) days in the placebo group (*P* = 0.54). The exploratory analysis with data from the first cold season including the participants with no colds resulted in a mean of 3.65 ± 5.29 days with symptoms in the probiotic group compared to 6.02 ± 7.64 days in the placebo group (*P* = 0.02).

### Analysis of immune markers

No differences were identified between the groups for any of the lymphocyte populations analyzed by flow cytometry (data not shown). However, in a subgroup analysis, a small but significant increase in the percentage of memory CD8+ cells (CD3+CD8+Foxp3-CD45R0+) could be observed in the probiotic group within the first 2 weeks of intervention for the subjects who did not get a cold (mean change of 1.9 ± 9.5; *P* = 0.048). The analysis of immune markers in serum revealed a significant difference between the 2 groups at 12 weeks for the change from baseline in absolute values of IFN-γ (*P* = 0.045), as presented in **Table 3**. For TNF-α, there was a significant reduction in absolute values from baseline to 12 weeks within the placebo group (*P* < 0.05) but not within the probiotic group. A difference between the groups was also seen for the absolute change in fractalkine from baseline to 12 weeks (*P* = 0.064). There were no differences identified between the 2 study groups for any of the other serum markers analyzed (i.e., IP-10, TRAIL, IFN-α2a, IL-2Rα; data not shown).

**TABLE 3:** Change in absolute values from baseline to 2 and 12 weeks of intervention

	Probiotic *n* = 29	Placebo *n* = 28	Estimated difference between groups (95% CI range)	*P* ^1^	Effect size
IFN-γ, pg/ml					
2 wks	−1.59 ± 8.01 (−2.50, 0.82)	1.90 ± 16.4 (−3.03, 1.67)	−2.2 (−7.40 to 2.99)	0.40	
12 wks	−3.11 ± 9.60 (−2.34, 0.63)	5.06 ± 20.8 (−2.48, 2.25)	−7.01 (−14.9 to 0.93)	0.045	0.50
TNF-α, pg/ml					
2 wks	−0.07 ± 0.41 (−0.17, 0.10)	0.06 ± 0.56 (−0.18, 0.14)	−0.078 (−0.275 to 0.120)	0.44	
12 wks	−0.06 ± 0.28 (−0.27, 0.15)	−0.14 ± 0.43^2^ (−0.24, 0.08)	0.13 (−0.017 to 0.281)	0.081	0.22
Fractalkine, pg/ml					
2 wks	−353 ± 1440 (−748, 357)	−136 ± 1280 (−725, 538)	0.015 (−514 to 514)	1.0	
12 wks	227 ± 1420 (−391, 817)	−395 ± 1390 (−1190, 632)	683 (−42.2 to 1410)	0.064	0.44

Change from baseline for serum IFN-γ, TNF-α, and fractalkine in the subgroup of participants that provided serum samples during the first cold season of the study (2013–2014), including subjects with and without colds. Values are means ± SDs (Q1, Q3).

^1^Significant difference between the groups was set at *P* < 0.05. A mixed model was used for the analysis of serum markers and comparison between the groups, whereas a Wilcoxon signed-rank test was applied for the analysis of the changes from baseline within each group.

2 Significant change from baseline at *P* < 0.05.

### Concomitant medication

The use of a concomitant medication was reported by 560/898 subjects (**Table 4**) and tended to be lower in the probiotic group (59.4%) compared to the placebo group (65.3%; *P* = 0.07). Analgesics were the most commonly reported concomitant medication, used by 26.3% of the subjects in the probiotic group and by 32.0% in the placebo group (*P* = 0.07). The difference between the groups was even bigger during the first winter season, with 21.1% (26/123) of the participants in the probiotic group using analgesics compared to 33.3% (42/126) in the placebo group (*P* = 0.045).

**TABLE 4:** Usage of concomitant medication

Concomitant medication	Probiotic, *n* (%)	Placebo, *n* (%)	*P*
Subjects using concomitant medication	266 (59.4)	294 (65.3)	0.07
Type of medication used			
Analgesics	118 (26.3)	144 (32.0)	0.07
Sex hormones and modulators of the genital system	85 (19.0)	97 (21.6)	0.36
Nasal preparations	41 (9.2)	49 (10.9)	0.44
Anti-inflammatory and antirheumatic products	42 (9.4)	46 (10.2)	0.74
Thyroid therapy	30 (6.7)	44 (9.8)	0.11
Agents acting on the renin-angiotensin system	30 (6.7)	35 (7.8)	0.61
Antibacterials for systemic use	21 (4.7)	24 (5.3)	0.76
Cough and cold preparations	26 (5.8)	19 (4.2)	0.29

Medication use during the intervention period of 12 weeks for the total length of the study (all three seasons 2013–2016), in the intent-to-treat population (including all subjects, irrespective of having reported a cold or not). Results show number and frequency of participants with self-reported usage of concomitant medication in each study group, *n* (%). Only medications with a usage frequency of at least 5% in the total study population (both study groups) were included in the analysis. There were *n* = 448 randomized subjects in the probiotic group and *n* = 450 in the placebo group. Fisher´s exact test was used for the comparison between the study groups.

### Safety

The numbers of reported adverse and severe adverse events did not differ between the groups, and most AEs were not related to the study product (**Table 5**). There were 7 AEs that led to study termination: 4 in the probiotic group (1 case of pain in the arm after blood sampling, 2 cases of headache, and 1 case of nausea) and 3 in the placebo group (1 case of acute gastritis and 2 cases with gastro-enteritis). Only a few cases were rated as being probably or possibly related to the study product, with no difference between the groups. The 2 AEs with probable relation to the probiotic product included flatulence of mild intensity and gastric disorders of moderate intensity, whereas the 7 AEs with possible relation to the probiotic included severe stomachache, headache of mild or moderate intensity, moderate stomach cramps, eructation of mild intensity, and 2 cases of nausea. In the placebo group, there were 8 subjects with AEs possibly related to the study product, and the events were of similar types as those reported in the probiotic group. There were 4 severe adverse events reported in total, with no difference between the groups and with no relation to the study product (Table 5).

**TABLE 5:** Adverse events and severe adverse events registered during the study

	Probiotic, *n* (%)	Placebo, *n* (%)
All subjects with AEs	188 (42.0)	184 (40.9)
Subjects with AEs not related to the study product	176 (39.3)	175 (38.9)
Subjects with AEs related to the study product	12 (2.7)	9 (2.0)
Definite^1^	0 (0)	0 (0)
Probable^1^	2 (0.4)	0 (0)
Possible^1^	7 (1.6)	8 (1.8)
Not assessable^1^	3 (0.7)	1 (0.2)
Subjects with SAEs	2 (0.4)	2 (0.4)

Data are in the intent-to-treat population (including all subjects irrespective of having reported a cold or not). Results show number of participants that reported adverse or severe adverse events in each study group and the corresponding frequency among randomized subjects in the respective group, *n* (%). Randomized subjects were *n* = 448 in the probiotic group and *n* = 450 in the placebo group. Abbreviations: AE, adverse event; SAE, severe adverse event.

^1^The association of AEs to the study product was either definite, probable, possible, or could not be assessed.

## Discussion

In the present study, we investigated the efficacy of *L. plantarum* HEAL9 and *L. paracasei* 8700:2 (Probi Defendum^®^) against community-acquired common-cold infections in healthy adults.

The severity of the colds did not differ between the study groups despite the trend seen in the first season for milder colds in the probiotic group compared to the placebo group. This trend was evaluated by an IDMC that recommended the extension of the recruitment phase to the following cold season. The post hoc exploratory analysis of the severity score at the group level (i.e., subjects without colds had a score of “0”) in the first cold season resulted in a significantly lower mean daily WURSS−21 symptom score in the probiotic group compared to the placebo group. The results were also significant for the cold-specific nasal, pharyngeal, and bronchial symptoms.

The study groups were similar in terms of incidence of the colds, but fewer subjects had recurrent infections in the active group (20.8%) compared to the placebo group (29.8%), leading to significantly fewer colds per person in the probiotic group for those participants who had reported at least 1 cold. The results from this study are consistent with the results from the study by Berggren et al. (18) that was conducted with the same probiotic product in Sweden during the cold season of 2006–2007, in which 33% of the participants in the control group had ≥2 episodes compared to 21% of the probiotic group (*P* = 0.024). When comparing the 2 studies, a higher incidence of colds overall in the control group was identified in the study by Berggren, with 67% of those participants experiencing at least 1 cold compared to 41.9% in the current study, indicating that 2006–2007 might have been a more severe common cold season in general. Another study that also evaluated the efficacy of the same probiotic strains on community-acquired common colds was conducted in Germany during the cold season of 2007–2008 (19). The incidence of at least 1 cold was 48% in the placebo group, with no difference compared to the probiotic group (46.5%; *P* = 0.65). Both Berggren et al. (18) and Busch et al. (19) reported a reduction in the number of days with cold symptoms for the participants who consumed *L. plantarum* HEAL9 and *L. paracasei* 8700:2. In the current study, this was only seen at a group level in the first cold season, with significantly fewer symptom days/person in the probiotic group compared to the placebo group (3.65 ± 5.29 days vs. 6.02 ± 7.64 days, respectively; *P* = 0.02), but there was no difference measured across all 3 seasons together. Based on the results of symptom severity and number of days with symptoms, the conclusion is that in the current study, which was conducted during 3 consecutive cold seasons, intake of the probiotic product had no direct effect on the severity or duration of the colds. Nevertheless, there was a significantly lower mean number of colds per person and an almost significant reduction in the frequency of recurrent cold infections in the participants who had at least 1 cold.

The probiotic benefit against infectious diseases can be supported by various mechanisms, either direct, such as competitive exclusion of the infectious agent from mucosal sites, or indirect, such as modulation of the immune response to the infection. Intake of *L. paracasei* 8700:2 at 10^10^ CFU/day for 14 days significantly improved in vitro phagocytic activity of polymorphonuclear cells and tended to increase the population of NKT cells in healthy adults (17). Furthermore, there was a tendency towards increased expression of the memory marker CD45R0 within the population of CD8+ T lymphocytes, which could be interpreted as a possible priming of the immune system. Although this effect on the CD8+ cells was not seen in the current study, there was a significant change over time for the percentage of CD8+CD45R0+ lymphocytes in the subgroup of participants who consumed probiotics and did not report any colds. It might be that the higher probiotic dose applied in Rask et al. (17) induced a faster and more potent immunological response that was not detectable with the dose applied in the current study and in the analysis within 2 weeks of intervention.

In the current study, the change in serum levels of IFN-γ from baseline to 12 weeks differed significantly between the 2 study groups. In the context of the reduced number of common colds/person with *L. paracasei* 8700:2 and *L. plantarum* HEAL9, we could cautiously interpret the results for IFN-γ as a reduced need in the probiotic group for a proinflammatory response involving cellular immunity or as an efficiently maintained balance between pro- and anti-inflammatory activities. An interesting difference between the study groups was also seen for the change in the levels of fractalkine from baseline to 12 weeks (*P* = 0.064; d = 0.44), although in opposite directions from the changes seen for IFN-γ. An hypothesis is that the above results show a trend in the probiotic group for proinflammatory priming of the immune system by fractalkine and a balancing effect of this activity from IFN-γ. Fractalkine is a chemokine that is involved in various inflammatory diseases. It is produced in the intestinal tract following a trigger by proinflammatory signals and may be involved in directing intestinal epithelial cell-lymphocyte interactions, as well as the attraction of lymphocytes into the intestinal lamina propria. Recently it was reported that fractalkine may be involved in both immunopathological and antiviral immune responses to rhinovirus infection (23). Surface expression of CX3CR1, the receptor for fractalkine, has been demonstrated in NK cells, monocytes, CD8+ T cells, and, to a lesser extent, CD4+ T cells. Tang et al. (24) reported that fractalkine is upregulated on the surface of airway epithelial cells upon infection with rhinovirus and exposure to IFN-γ. In this case, the production of fractalkine probably represents the proinflammatory response that sets off the antiviral host activity required for the eradication of the infection. Nasal epithelial cells from asthmatic subjects were found to produce more fractalkine than those from nonasthmatic subjects, which indicates that fractalkine may play a role in asthma exacerbations (24). It is obvious that in the case of asthma, the excessive production of fractalkine becomes a problem, and there seems to be no balance between the pro- and anti-inflammatory activity linked to the viral infection. In view of the results obtained in the current study, though, it could be hypothesized that the small induction of fractalkine by the probiotics might contribute to the priming of the immune system in a controlled manner, since the increase in fractalkine was not followed by an increase in IFN-γ. The probiotic impact on the levels of fractalkine and IFN-γ was only seen after 12 weeks and not after 2 weeks of intervention. We may speculate that the absence of a measurable probiotic impact on the cellular immune markers analyzed in the study at baseline and after 2 weeks of intervention could be explained by the early time point chosen for the analysis (i.e., 2 weeks after the start of the intervention).

As already discussed, the reduced severity of common colds by *L. plantarum* HEAL9 and *L. paracasei* 8700:2 has previously been documented both in adults and children (18–20). In Berggren et al. (18), intake of the probiotic was associated with a significant reduction in the incidence and duration of cold episodes, as well as reduced severity of pharyngeal symptoms. In the follow-up study by Busch et al. (19), the probiotic significantly reduced the duration and severity score of the reported cold episodes but had no impact on the incidence rate. When the same combination of strains was evaluated in children attending day care, there was a significant reduction in the severity of “runny nose” and of concomitant medication use (20). The varied incidence rates of reported colds in the control groups in these studies, as well as the difficulties in repeating and confirming the probiotic benefit on all aspects of a common cold (i.e., incidence, frequency, duration, severity) are indicative of the difficulties faced in seasonal studies with factors that cannot be controlled.

Common colds are viral infections of the upper respiratory tract, usually caused by rhinoviruses. It has been reported that virus-virus interactions may impact the population dynamics of influenza with the common cold, leading to less frequent colds during intensive flu seasons. Based on information extracted from the European Centre for Disease Prevention and Control (https://www.europa.eu), the flu season in Germany during 2013–2014 was of low intensity compared to the following seasons of 2014–2015 (high intensity) and 2015–2016 (low to medium intensity). In the current study, the heterogeneity among the 3 cold seasons is reflected in the higher incidence of reported cold episodes in the control group during 2013–2014 compared to the other 2 seasons. Although it is difficult to explain the heterogeneity observed, it can be seen as a strength of the study that there was a significant probiotic effect on the mean number of colds per person despite the variation in the 3 cold seasons.

There are other examples of probiotic bacteria that have been evaluated for their immunomodulatory efficacy in common colds. Zhang and colleagues (25) randomized 136 young Chinese adult volunteers who had experienced 4 or more cold episodes in the past year to 150 ml per day of either a placebo drink or a triple-strain probiotic drink (total of approximately 10^10^ CFU/day for 12 weeks). Consumption of the probiotics significantly reduced the incidences of URTI and flu-like symptoms, compared to consumption of placebo, and there were significantly higher levels of IFN-γ in serum and secreted IgA in the gut. *Lactobacillus rhamnosus* GG and *Bifidobacterium animalis* ssp *lactis* BB12 were evaluated in a study with military conscripts on the seasonal occurrence of upper respiratory and gastrointestinal infections (26). There was no effect on the symptom incidence rate and duration, but there were significant reductions of some respiratory tract symptoms after intake of the probiotic, as compared to intake of the placebo, for 150 days, although not after 90 days. It might be that the longer intervention period was needed for more efficient modulation of the immune system or for the experience of more colds. In comparison, the same combination of strains was also evaluated in a study with 231 college students in the United States (27). Daily intake of the probiotic product with a least 1 × 10^9^ bacteria per strain for 12 weeks significantly reduced the duration and WURSS−21 severity score of the reported URTIs, as well as the number of missed school days, compared to intake of placebo. Based on the above studies and other publications within the field, it becomes obvious that there are variations among strains with regard to their immunomodulatory capacity and that there are aspects, such as the dose and the length of intervention, that may impact the results. Moreover, seasonal factors may also undermine the interpretation of the data obtained.

To conclude, *L. plantarum* HEAL9 and *L. paracasei* 8700:2 have repeatedly been shown to be well tolerated and to reduce the incidence, duration, and severity of common colds both in adults and children. Existing data indicate that these bacteria may prime the immune system in a protective manner towards future infections of common colds. In the current study, intake of the probiotic product reduced the frequency of recurrent cold infections and resulted in a significantly lower mean number of colds in the participants with colds. However, the exact mechanism underlying the probiotic efficacy and the understanding of what parameters may impact this remain to be untangled.

## Notes

This research was funded by Probi AB.

Author disclosures: ILA, EAN, NL, and TMN are employed by Probi AB, which is a research and B2B probiotics company. The other author reports no conflict of interest.

Abbreviations used: AE, adverse events; DSM, Deutsche Sammlung von Mikroorganismen; IDMC, independent data monitoring committee; ITT, intent to treat; NKT, natural killer T cells; iNKT, invariant NKT cells, URTI, upper respiratory tract infection; WURSS-21, Wisconsin Upper Respiratory Symptom Survey−21.
